# Editorial Board Collection Series: Insight into Neurosurgery

**DOI:** 10.3390/brainsci16060622

**Published:** 2026-06-10

**Authors:** Francesco Signorelli, Massimiliano Visocchi

**Affiliations:** Department of Neurosurgery, Fondazione Policlinico Universitario “A. Gemelli” IRCCS, Catholic University, Largo Agostino Gemelli, 800168 Rome, Italy; massimiliano.visocchi@unicatt.it

The present Special Issue brings together five contributions that reflect the evolving landscape of modern neurosurgery, where technological innovation, multidisciplinary integration, and individualized patient care increasingly define clinical decision-making. Although the contributions address distinct pathological entities—from skull-invasive tumors and spinal oncology to idiopathic normal pressure hydrocephalus (iNPH), cervical spine complications, and traumatic brain injury (TBI)—they all converge on a common objective: improving patient outcomes through more precise diagnosis, safer surgical strategies, and better long-term functional recovery.

A first major theme to emerge from this collection is the growing role of advanced technologies in surgical planning and postoperative management. The study on skull-invasive tumors (Contribution 1) highlights how one-step cranioplasty combined with tumor resection can be further optimized through the integration of Mixed Reality (MR). While conventional neuronavigation remains superior in terms of intraoperative precision, MR demonstrates substantial value in preoperative planning, anatomical understanding, and patient–family communication. This work underlines an important concept in contemporary neuro-oncology: innovation does not necessarily replace established standards but can serve as a complementary tool that enhances the overall surgical workflow.

Similarly, the review on carbon fiber-reinforced polyetheretherketone (CFR-PEEK) implants in spine tumor surgery (Contribution 2) addresses a critical challenge in multidisciplinary oncological care: the interaction between surgical reconstruction and adjuvant radiotherapy. Traditional titanium instrumentation, while mechanically reliable, often limits postoperative imaging interpretation and radiation planning because of significant artifacts. CFR-PEEK implants offer a promising alternative by improving radiological visualization and facilitating safer, more effective radiation delivery. This article emphasizes how implant selection itself can become a strategic oncological decision, extending beyond mechanical stabilization toward optimizing the entire treatment pathway.

Another central topic of this issue is the refinement of treatment strategies for cerebrospinal fluid disorders, particularly iNPH. The ENVENTOR-iNPH protocol (Contribution 3) proposes an ambitious multicenter randomized comparison between ventriculoperitoneal shunting (VPS), the current gold standard, and endoscopic third ventriculostomy (ETV), a less invasive alternative that may offer fewer complications. Importantly, the study expands outcome assessment beyond traditional clinical scales by incorporating advanced neuroimaging, navigated transcranial magnetic stimulation, and wearable motion analysis. This multidimensional approach reflects the broader shift in neurosurgery toward functional and patient-centered outcome evaluation rather than purely technical success. If successful, this trial may significantly influence future treatment algorithms for selected iNPH patients.

This issue also addresses the importance of recognizing and preventing rare but clinically significant surgical complications. The systematic review and case reports on hypoglossal nerve palsy following cervical spine surgery (Contribution 4) provide valuable insights into an uncommon yet potentially disabling complication. By combining literature review with illustrative clinical cases, the authors reinforce the importance of anatomical knowledge, careful positioning, and intraoperative vigilance. Such contributions are particularly valuable because uncommon complications are often underrecognized despite their impact on postoperative quality of life. Prevention remains the most effective strategy, and awareness is the first step toward prevention.

Finally, the scoping review on diffusion tensor imaging (DTI) in traumatic brain injury (Contribution 5) reinforces the expanding role of advanced neuroimaging as both a diagnostic and prognostic tool. Conventional imaging often underestimates the burden of diffuse axonal injury and microstructural white matter damage. DTI offers a more sensitive method for detecting these alterations and demonstrates promising predictive value for neurological outcomes. As TBI management increasingly focuses on long-term recovery and rehabilitation planning, the ability to better stratify patients through imaging biomarkers may become essential for personalized treatment strategies.

Taken together, these five articles illustrate how contemporary neurosurgery is moving beyond isolated operative interventions toward integrated treatment ecosystems in which surgery, imaging, biomaterials, neurophysiology, and rehabilitation are deeply interconnected. Precision medicine in neurosurgery is no longer limited to technical excellence in the operating room; it increasingly involves selecting the right technology, the right implant, the right timing, and the right multidisciplinary pathway for each individual patient.

This Special Issue also highlights an important methodological evolution: the growing emphasis on prospective protocols, systematic reviews, and translational technologies capable of bridging clinical practice and research. Such approaches are essential for generating stronger evidence in fields where decision-making has often relied on institutional experience and expert opinion.

In conclusion, the contributions collected here provide not only valuable updates across several subspecialties of neurosurgery but also a broader vision of where the field is heading. Innovation must remain clinically meaningful, technology must serve patient-centered goals, and multidisciplinary collaboration must guide increasingly complex therapeutic decisions. These principles represent the true foundation of progress in modern neurosurgical care.

